# Identification of Several Mutations in *ATP2C1* in Lebanese Families: Insight into the Pathogenesis of Hailey-Hailey Disease

**DOI:** 10.1371/journal.pone.0115530

**Published:** 2015-02-06

**Authors:** Waed Btadini, Ossama K. Abou Hassan, Dana Saadeh, Ossama Abbas, Farah Ballout, Abdul-Ghani Kibbi, Ghassan Dbaibo, Nadine Darwiche, Georges Nemer, Mazen Kurban

**Affiliations:** 1 Department of Dermatology, American University of Beirut, Beirut, Lebanon; 2 Department of Biochemistry and Molecular Genetics, American University of Beirut, Beirut, Lebanon; 3 Department of Dermatology, Columbia University, New York, New York, United States of America; 4 Department of Pediatrics, American University of Beirut, Beirut, Lebanon; St. Georges University of London, UNITED KINGDOM

## Abstract

**Background:**

Hailey-Hailey disease (HHD) is an inherited blistering dermatosis characterized by recurrent erosions and erythematous plaques that generally manifest in intertriginous areas. Genetically, HHD is an autosomal dominant disease, resulting from heterozygous mutations in *ATP2C1*, which encodes a Ca^2+^/Mn^2+^ATPase. In this study, we aimed at identifying and analyzing mutations in five patients from unrelated families diagnosed with HHD and study the underlying molecular pathogenesis.

**Objectives:**

To genetically study Lebanese families with HHD, and the underlying molecular pathogenesis of the disease.

**Methods:**

We performed DNA sequencing for the coding sequence and exon-intron boundaries of *ATP2C1*. Heat shock experiments were done on several cell types. This was followed by real-time and western blotting for ATP2C1, caspase 3, and PARP proteins to examine any possible role of apoptosis in HHD. This was followed by TUNEL staining to confirm the western blotting results. We then performed heat shock experiments on neonatal rat primary cardiomyocytes.

**Results:**

Four mutations were detected, three of which were novel and one recurrent mutation in two families. In order for HHD to manifest, it requires both the genetic alteration and the environmental stress, therefore we performed heat shock experiments on fibroblasts (HH and normal) and HaCaT cells, mimicking the environmental factor seen in HHD. It was found that stress stimuli, represented here as temperature stress, leads to an increase in the mRNA and protein levels of ATP2C1 in heat-shocked cells as compared to non-heat shocked ones. However, the increase in ATP2C1 and heat shock protein hsp90 is significantly lower in HH fibroblasts in comparison to normal fibroblasts and HaCaT cells. We did not find a role for apoptosis in the pathogenesis of HHD. A similar approach (heat shock experiments) done on rat cardiomyocytes, led to a significant variation in ATP2C1 transcript and protein levels.

**Conclusion:**

This is the first genetic report of HHD from Lebanon in which we identified three novel mutations in *ATP2C1* and shed light on the molecular mechanisms and pathogenesis of HHD by linking stress signals like heat shock to the observed phenotypes. This link was also found in cultured cardiomyocytes suggesting thus a yet uncharacterized cardiac phenotype in HHD patients masked by its in-expressivity in normal health conditions.

## Introduction

Hailey-Hailey disease (HHD; OMIM 16960), also known as familial benign pemphigus, is a rare autosomal dominant genodermatosis that was initially described by the Hailey brothers in 1939[1]. HHD has variable expressivity and complete penetrance, and usually presents around the third or fourth decade of life and lasts months to years[2]. Clinical features are characterized by recurrent painful grouped vesicles, pustules and plaques that occur in intertriginous areas including the neck, the axilla, the groin and the perineum. These eventually progress to severe and painful fissures and erosions that can be physically and psychologically debilitating. Histologically, HH is characterized by suprabasilar acantholysis and blister formation secondary to desmosomal abnormalities in a pattern resembling a “dilapidated brick wall”[2].

HHD is caused by mutations in the *ATP2C1* gene. *ATP2C1* encodes for a p-type Ca^2+^-transport ATPase, which is part of the Ca^2+^-transport ATPases (SpCA) secretory-pathway and is expressed on the *trans*-golgi apparatus[3]. This manganese-dependent enzyme is involved in Ca^2+^ homeostasis. It catalyzes the hydrolysis of ATP coupled with the transport of calcium and manganese ions from the cytosol to the lumen of the Golgi apparatus. In wild type keratinocytes, adequate Golgi Ca^2+^homeostasis is required for the proper functioning of the Golgi and in particular for posttranslational protein-modifications. In HHD, heterozygous mutations in *ATP2C1* disrupt Ca^2+^ homeostasis leading to decreased Golgi Ca^2+^ stores and increased cytosloic Calcium[4] This in turn negatively affects Ca2^+^dependent proteins; including those belonging to the cadherin family[5] Such proteins include desmogleins and desmocollins which assemble together and with other proteins to form the desmosomes. Therefore, mutations in *ATP2C1* would compromise the desmosomal assembly leading to HHD.

Several external factors contribute to the pathogenesis of HHD. These include increased humidity, heat, sweat and increased body temperature. This explains why individuals show clinical manifestations mainly in intertriginous areas and have exacerbation of their condition during hot seasons.

In this study, we aimed at identifying and analyzing underlying mutations in five patients from unrelated families diagnosed clinically and histologically with HHD. We, then, tried to mimic external factors by subjecting and comparing cells from affected individuals with unaffected ones by performing heat shock experiments. We investigated whether apoptosis contribute to the pathogenesis of HHD. Finally, since ATP2C1 is expressed in several mammalian tissues [6], we studied the expression of ATP2C1 in rat cardiomyocytes under normal conditions and after performing heat shock experiments in light of numerous studies showing that abnormalities in Calcium handling proteins could be associated to numerous cardiac pathologies including conduction system and heart failure[7]. The most striking example being the mutations in ATP2A2, which are responsible for the Darier’s disease[8].

## Materials and Methods

### 2.1. Subjects and mutation analysis of *ATP2C1* gene

Five Lebanese patients were recruited all of whom were clinically and histologically diagnosed with HHD at the American University of Beirut Medical Center (AUBMC). All of the patients presented were in the third or fourth decade of life showing blisters and erosions affecting the armpits and the inguinal regions and nape of neck, consistent with HHD. A positive family history for HHD was recorded in all of the cases. After obtaining written informed consents, we collected peripheral blood sample from the family members and unrelated healthy control individuals in EDTA-containing tubes. Genomic DNA was isolated from peripheral blood lymphocytes according to standard extraction techniques. The study was approved by the Ethics Committee of the American University of Beirut Medical Center under protocol number (DER.MK.01), and in adherence to the declaration of Helsinki Principles. All exons and exon-intron boundaries for ATP2C1 were performed. The amplified PCR products were directly sequenced in an ABI 3100 Genetic Analyzer using the ABI Prism Big Dye Terminator Cycle Sequencing Ready Reaction Kit (PE Applied Biosystems, USA). The results were analyzed using the Applied Biosystems software and compared to databanks on the UCSC browser.

### 2.2. Reverse transcription and cDNA sequencing

Total RNA was extracted from human fibroblasts obtained from punch biopsies using Nucleospin RNAII kit (Macherey-Nagel) in accordance with the manufacture’s protocol. cDNA synthesis was carried out using RevertAid First strand cDNA synthesis kit (Thermo Scientific) according to the manufacturer’s instructions. cDNA amplification of patient 3 was carried out using the forward primer 5’ CCTTATTATGCTGCTTCTGG 3’ and the reverse primer 5’ CTTTGCTTTGCCACATCTGA 3’) and amplified the region spanning exons 4 to 8. cDNA amplification of patient 4 was carried out using the forward primer 5' GAAGAAAAGGGCCATTGTGA 3' and the reverse primer 5' CATGCGTGCCTTCTCTTGT 3', and amplified the sequence spanning exons 12 to 17. cDNA sequencing was then performed using the forward primers already mentioned by Sanger ABI 3100 Genetic Analyzer.

### 2.3. Cell culturing

Fibroblasts were grown from punch biopsies in Dulbecco’s Modified Eagle Medium (DMEM-LONZA, USA). Biopsies were first cut by a surgical scalpel into 4–5 pieces. The latter were then collected with 5ml DMEM and centrifuged. The resulting pellet was then distributed into 6-well plate with 3 ml DMEM supplemented with 20% heat inactivated FBS (fetal bovine serum) and 1% penicillin-streptomycin. As for HaCaT cells, they were grown up to passage 7 in DMEM supplemented with 10% heat-inactivated FBS, 1% penicillin-streptomycin, and 1% sodium pyruvate.

Cardiomyocytes were prepared from rats of 1–4 days of age that were decapitated, and the hearts were removed and placed in MEM-Joklik (SIGMA-Aldrich, USA). Blood clots were removed from the hearts which were later minced and digested with collagenase in MEM-Joklik. Cells from the four subsequent digestions (20 min each) were collected in 50-ml sterile tubes containing 10ml FBS, and sedimented by centrifugation at 500×g for 5 minutes at room temperature. The cell pellets, which consisted of cardiomyocytes and fibroblasts, were resuspended in 30 ml DMEM with 10% FBS. The cell suspensions were then filtrated with 100μm filter. The primary culture was cleaned up of adherent cells like fibroblasts by two cycles of 30 minutes preplatting at 37°C in 150 mm dishes. Cardiomyocyte-enriched suspensions were collected from the culture dishes in a 50ml sterile tube and centrifuged 500×g for 5 minutes at room temperature. The resulting pellet, mainly consisting of cardiomyocytes was resuspended in 30ml DMEM with 10% FBS and the cells were seeded in a 100 mm petriplate (4*10^6^ cells/plate). The second day after plating the culture medium was replaced for serum free hormone free media. Cardiomyocytes were maintained in culture two days before conducting the experiment. Animal work was approved by the American University of Beirut Institutional Animal Care and Use Committee (IACUC 001/05). All animals received care in accordance with approved institutional animal care guidelines and according to the Guide for the Care and Use of Laboratory Animals of the National Academy of Science and US National Institutes of Health guidelines.

### 2.4. Heat shock experiments

In order to mimic one of the environmental factors encountered in HHD, heat-shock experiments were conducted. Fibroblasts (between passages 4 and 7) were left for 40 minutes at 43°C in a CO_2_ incubator, and then transferred to a 37°C CO_2_ incubator so that they restore their normal gene expression profiles. mRNA or proteins were extracted 160 minutes later. As for primary cardiomyocytes, a series of heat shock experiments were performed at 20 and 30 minutes.

### 2.5. Quantitative Real time PCR

Quantitative real-time PCR was conducted using QuantiTect SYBR Green PCR kit (Qiagen, USA). The expression primers used to detect the effect of heat shock on ATP2C1 expression were the following: ATP2C1: Forward primer 5’ CACTAAGTTCCAGATCCCA 3’ and reverse primer 5’-ATGCTTAGGCTCTCAGTCT-3’; Hsp90: Forward primer 5-TCTGGAAGATCCCCAGACAC-3’ and reverse primer 5’-AGTCATCCCTCAGCCAGAGA-3'. Rat ATP2C1: Forward primer 5'-GCCTTGGAGTAGAGCCAGTG-3’ and reverse primer 5’-TGTTGTGTCTCGGGGTGTTA-3’. Rat hsp90: Forward primer 5’-TGAAGGAATTTGAGGGCAAG-3’ and reverse primer 5’-CACCAATCGGTTTGACACAA-3’. GAPDH was used as an internal loading control and amplified using the following primers: Human GAPDH: Forward primer 5’-CCACCCAgAAgACTgTggAT-3’ and reverse primer 5’-TTCAgCTCAgggATgACCTT-3’; rat GAPDH: Forward primer: 5’-AGCCAAAAGGGTCATCATCATCT-3’, and reverse primer: 5’-GGGGCCATCCACAGTCTTCT-3’. Relative transcript abundance was normalized to the amount of GAPDH and quantitated by the 2^−ΔΔCT^ method (Table 1 list all real time PCR primer sequences).

**Table 1:** Real Time PCR primers.

Primer	Sequence
Human ATP2C1 F	5' CACTAAGTTCCAGATCCCA 3'
Human ATP2C1 R	5' ATGCTTAGGCTCTCAGTCT 3'
Human Hsp90 F	5' ATGCTTAGGCTCTCAGTCT 3'
Human Hsp90 R	5' AGTCATCCCTCAGCCAGAGA 3'
Rat ATP2C1 F	5' GCCTTGGAGTAGAGCCAGTG 3'
Rat ATP2C1 R	5' TGTTGTGTCTCGGGGTGTTA 3'
Rat Hsp90 F	5' TGAAGGAATTTGAGGGCAAG 3'
Rat Hsp90 R	5' CACCAATCGGTTTGACACAA 3'
Human GAPDH F	5' CCACCCAGAAGACTGTGGAT 3'
Human GAPDH R	5' TTCAGCTCAGGGATGACCTT 3'
Rat GAPDH F	5' AGCCAAAAGGGTCATCATCATCT 3'
Rat GAPDH R	5' GGGGCCATCCACAGTCTTCT 3'

### 2.6. Western blotting

Proteins were extracted from cells with Laemmli sample buffer (Biorad). Equal amounts of each extract were electrophoresed in 10% sodium dodecyl sulfate-polyacrylamide (SDS) gel electrophoresis, and then transferred into nitrocellulose membranes. Membranes were blocked for 1.5 hours with fat free milk 5%. After washing, membranes were blotted with primary antibodies overnight. We used a 1/500 dilution for the primary antibodies anti-ATP2C1, anti caspase-3, anti-PARP (Santa Cruz Biotechnology, Santa Cruz, Germany) and anti-GAPDH (Cell Signaling). Membranes were then blotted with corresponding secondary antibodies (1:5000 dilution) for 2 hours and developed prior to adding Luminol reagent (Santa Cruz Biotechnology, Santa Cruz, Germany). The density of the bands was then revealed using image J software. Relative quantification was carried out by normalizing the relative densities of the desired protein bands to the densities of their corresponding loading control band.

### 2.7. TUNEL staining

Paraffin-embedded tissues from HH patients and normal individuals were dewaxed and rehydrated through a graded series of xylene/ethanol treatments. Antigen retrieval was carried out by transferring the slides into sodium citrate buffer (pH 6) and irradiating them three times at 140 W in the microwave for three minutes each. Slides were immersed for 30 minutes in Tris-HCL, 0.1 M pH 7.5; containing 3% BSA and 20% normal bovine serum. Slides were then rinsed with PBS and incubated for 60 minutes in a humidified atmosphere at 37°C with 50 ul of TUNEL reaction mixture. For the positive control, tissues were incubated in DNase for 30 minutes prior to addition of TUNEL mix. Apoptosis, as judged by TUNEL positivity was then evaluated under fluorescence microscope.

## Results

### 3.1. Four mutations were detected in *ATP2C1* that cause Hailey-Hailey disease in Lebanese families

Five patients diagnosed with HHD of unrelated Lebanese families were genetically screened for mutations in *ATP2C1*. The entire coding sequence and the exon-intron boundaries of *ATP2C1* were amplified. Four different mutations were detected and are summarized in table 2. R39X, a previously reported nonsense mutation leading to the formation of a truncated protein, was detected in two of the families. Three novel mutations were also detected: An acceptor splice site mutation (r.313–1G>A), a missense mutation (c.1372G>C), and a deletion mutation (c. 2529_2532 del TTGT).

**Table 2:** Identification of three novel and one recurrent mutation in *ATP2C1* in Lebanese families with Hailey-Hailey disease.

Family	Mutation	location	Status
1	Nonsense R39X	Exon 3	Previously reported
2	Frame-shift mutation c. 2529_2532 delTTGT	Exon 24	Novel
3	Splice site mutation r.313–1G>A	Intron 5	Novel
4	Missense mutation c.1372G>C	Exon 14	Novel
5	Nonsense R39X	Exon 3	Previously reported

### 3.2. Analysis of the splice site and the missense mutation

**Fig 1:**
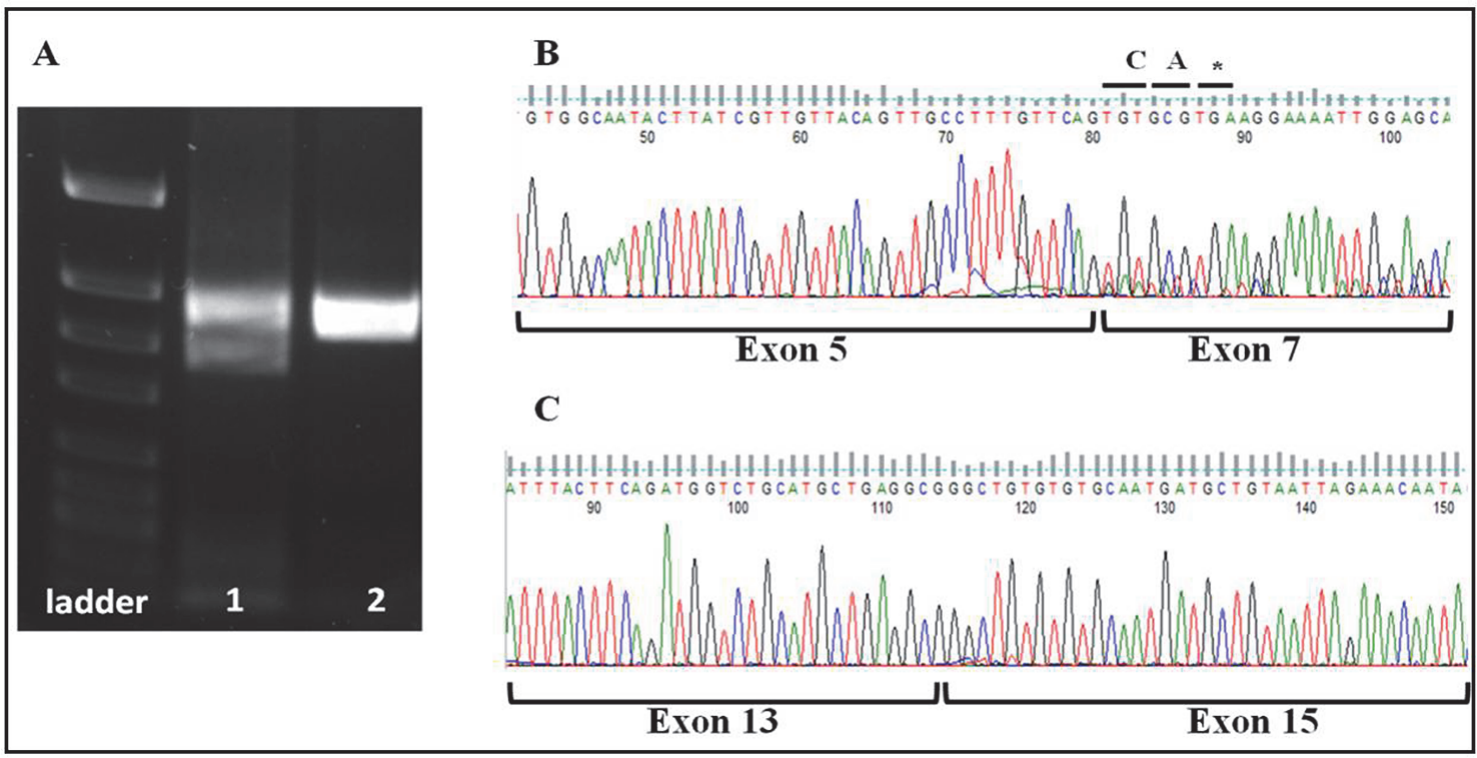
Analysis of the acceptor splice-site mutation and the missense mutation. (A) Agarose gel electrophoresis of cDNA amplified from the patient with r.313–1G>A (lane 1) and cDNA of normal individual (lane 2). The first lane reveals two bands indicating expression from two alleles; the normal (upper band) and the mutant (lower band). (B) cDNA sequence of patient with r.313–1G>A revealing skipping of exon 6, and resulting in a premature stop codon. (C) cDNA sequence of patient with (c.1372G>C) mutation revealing the skipping of exon 14.

In order to investigate the effect of the splice site mutation (r.313–1G>A) and the missense mutation (c.1372G>C) on alternative splicing, we amplified the cDNA in the region flanking the mutations from both sides. The splice site mutation occurs in intron 5, so the cDNA was amplified from exons 4 to 8. Skipping of exon 6 was validated by the two bands that appeared on gel (Fig. 1A) and by the cDNA sequencing results (Fig. 1B). The (r.313–1G>A) mutation not only causes the skipping of exon 6 but also generates a premature stop codon at the beginning of exon 7 (Fig. 1B), leading to a truncated protein. As for the missense mutation (c.1372G>C) which occurs in the last nucleotide of exon 14, we expected that it would affect the splice site and therefore we sequenced the cDNA in the region spanning exon 12 to 17. This revealed the skipping of exon 14 (in frame) (Fig. 1C) without any effect on the termination site proving that the missense variant is in fact an in-frame variant affecting splicing and therefore would be referred to r.1372G>C.

### 3.3. Heat-shock induces overexpression of *ATP2C1*

**Fig 2:**
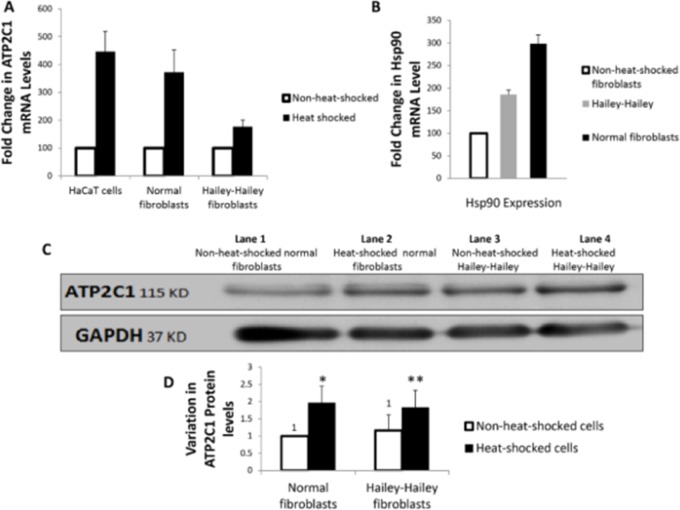
Heat-shock induces overexpression of ATP2C1. (A) Real-time PCR effects of heat-shock on HaCaT cells (p value = 0.004), fibroblasts from HH patient (p value = 0.003), and normal fibroblasts (p value = 0.01). (B) Hsp90 variation in expression before and after heat-shock in different cell types. (C) Western blot results; lane 1: non-heat-shocked normal fibroblasts, 2; heat-shocked normal fibroblasts, 3; non-heat-shocked Hailey-Hailey fibroblasts, 4; heat-shocked Hailey-Hailey fibroblasts. (D) ATP2C1 protein quantification (using image J software) based on the densities of the bands over GAPDH obtained through western blotting showing significant increase in normal fibroblasts (* p value = 0.01), and non-significant increase in HH fibroblasts (** p >0.05).

Symptoms of HHD mostly manifest in intertriginous areas, and are usually aggravated by sweat, friction and heat. Based on this, we conducted heat-shock experiments on different cell types (fibroblasts and HaCaT) to monitor the effect of stress on *ATP2C1* mRNA and protein expression. Real-time PCR experiments demonstrated an increase in *ATP2C1* expression in all of the different cell types (normal fibroblasts, HH fibroblasts and HaCaT cells). However; this increase was significantly lower in HH fibroblasts (Fig. 2A). Hsp90 increase in mRNA expression was used as a reference for an efficient heat-shock experiment. The increase in Hsp90 was also less witnessed in HH fibroblasts as compared to the other cells (Fig. 2B). In accordance with the real-time PCR results, western blotting revealed a slight increase in ATP2C1 protein levels after heat-shock (Fig. 2C-D).

### 3.4. Acantholysis is not attributed to apoptosis in Hailey-Hailey disease

**Fig 3:**
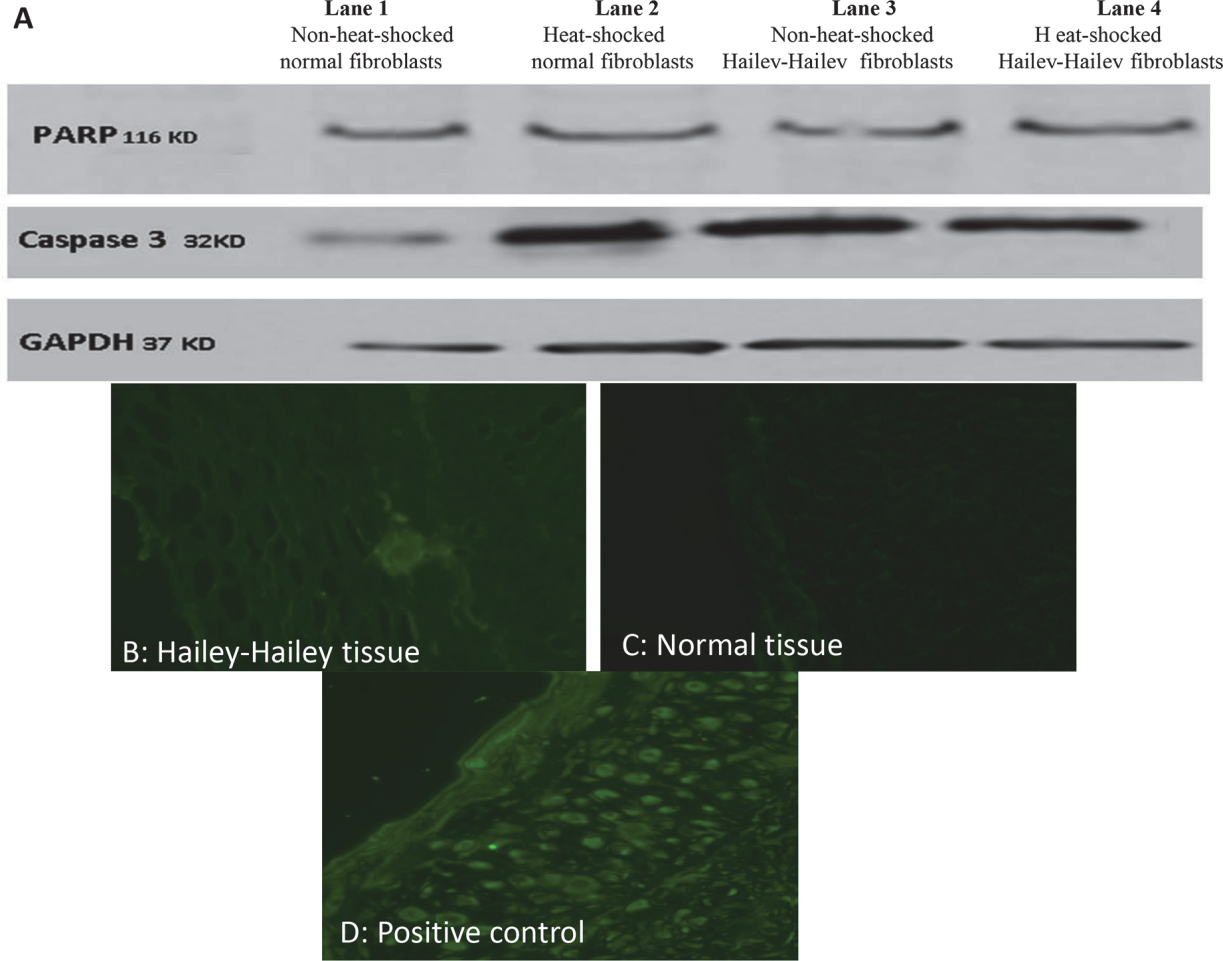
Investigating the role of apoptosis in Hailey-Hailey disease (A) Western blotting against caspase 3 and PARP proteins; lane 1: non-heat-shocked/control normal fibroblasts, lane 2: heat-shocked normal fibroblasts, lane 3: non-heat-shocked Hailey-Hailey fibroblasts, lane 4: Heat-shocked Hailey-Hailey fibroblasts. (B) TUNEL staining on paraffin-embedded tissues taken from affected area of a Hailey-Hailey patient. (C) Normal tissue negative for the TUNEL stain, (D) Positive control revealing apoptotic cells after treatment with DNase.

Heat-shock proteins are well known to inhibit apoptosis. Their weak elevation after stress in HHD led us to investigate whether apoptosis contributes to the pathogenesis of the disease. We performed western blotting for caspase 3 and PARP. No protein cleavage between these two proteins was detected indicating the absence of caspase-dependent apoptosis (Fig. 3A). To validate the absence of apoptosis, we conducted TUNEL staining on paraffin-embedded tissues taken from a patient with HHD and a normal individual. The TUNEL stain was negative in HH (Fig. 3B), and from normal tissues (Fig. 3C) as opposed to the positive control (Fig. 3D).

### 3.5. A plausible protective role of *ATP2C1* in the hearts of rats

**Fig 4:**
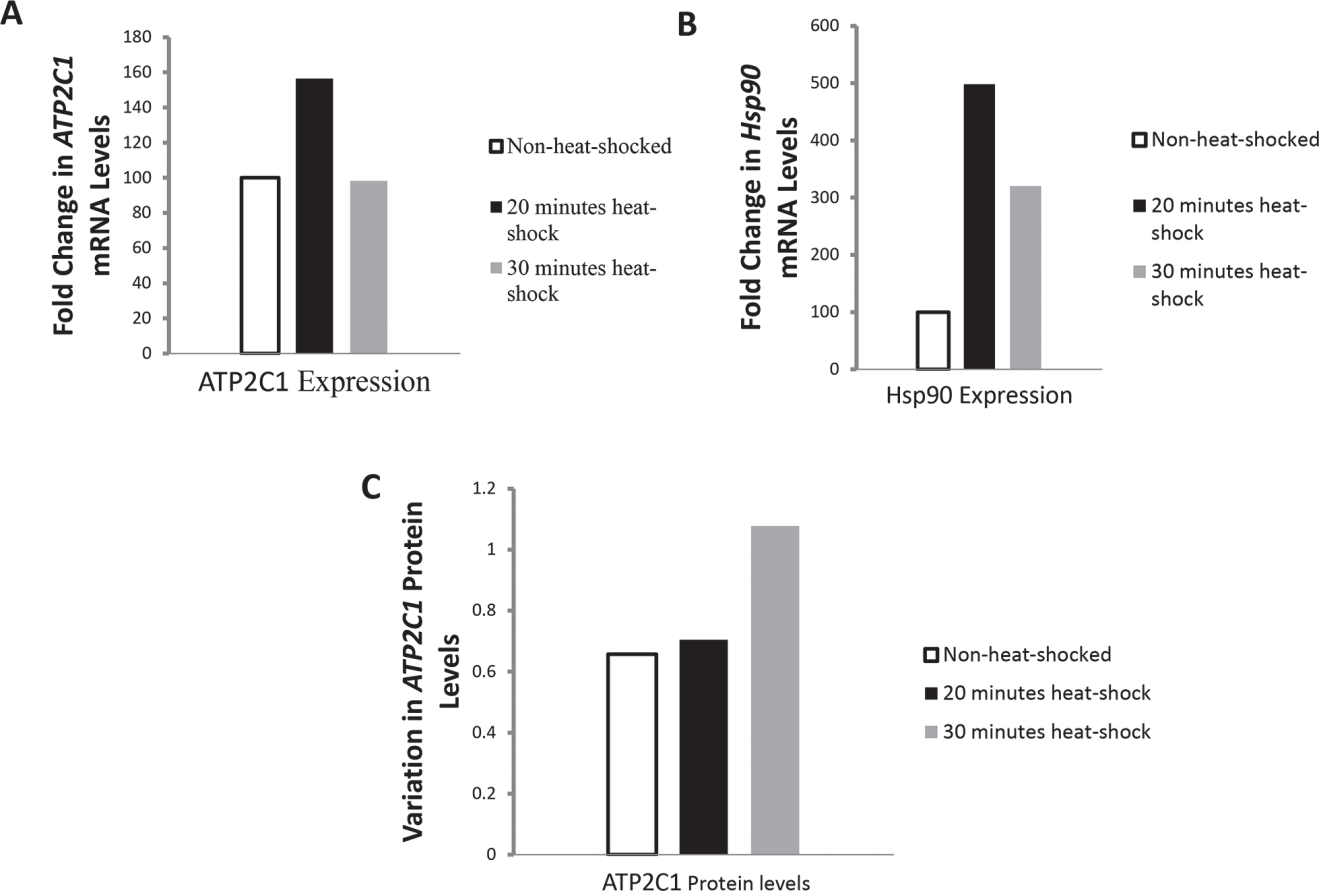
Effect of heat-shock on cardiomyocyte-ATP2C1 levels. (A) Fold change in ATP2C1 mRNA levels before and after heat-shock. (B) Fold change in Hsp90 mRNA levels before and after heat shock. (C) Variation in ATP2C1 protein levels before and after heat-shock.

We hypothesized that *ATP2C1* might have a possible function in the heart. It is unknown till now what effect could ATP2C1 defects exert at the level of the heart. We conducted heat-shock experiments on cardiomyocytes from rats at two time points (20 and 30 minutes). A significant variation in *ATP2C1* mRNA levels was detected, where these levels increased after 20 minutes of heat shock to decrease again at 30 minutes (Fig. 4A, B). However at the protein levels, western blotting revealed low ATP2C1 protein levels at 20 minutes and then an increase at 30 minutes of heat-shock suggesting an expected delay between the increase in mRNA and subsequent increase in protein (Fig. 4A-C).

## Discussion

ATP2C1 is a Ca^2+^/Mn^2+^ transporter, which regulates the concentrations of calcium and manganese in the lumen of Golgi apparatus. Since the Golgi is the site of protein processing and targeting, any perturbation in this complex may have adverse effects on the physiological cell functioning.

In this work we studied five Lebanese individuals from five different families with HHD. Among these families, two of them had a previously reported nonsense mutation p.R39X (c.317CGA>TGA). It is likely that this site represents a hotspot for mutations as this mutation has been previously reported in different families worldwide[9], as well as occurring at a CpG site which increases the possibility of mutations[10].

A combination of genetic and environmental factors, are essential for the manifestation of HHD. In order to mimic the environmental factors in our *in vitro* studies we performed heat-shock experiments and studied the effect of ATP2C1 on several cell types. The stress factor in our experiments promoted the increase in *ATP2C1* transcript and protein levels. These results are indicators of the involvement of ATP2C1 in homeostatic mechanisms. ATP2C1 level were reduced in HH fibroblasts, as compared to other cells, suggesting a certain disruption in the stress response of these cells. ATP2C1 levels increased, as heat-shock protein Hsp90 levels did in response to heat-shock, further suggesting a possible protective role of this protein in stressful situations.

We, then, investigated whether apoptosis plays a role in the initiation of the pathogenesis of HHD. In *C.elegans*, it was shown in an orthologue of *ATP2C1* (PMR1 ATPase) that a rise in HSP16.1, a heat shock protein localizing to the Golgi, results in inhibition of necrosis and cell death[11].

Conflicting results on the possible role of apoptosis in the pathogenesis of HHD and pemphigus vulgaris, an overlapping clinical and histological condition, have been reported[12,13]. Very recently, it was shown that the pathogenesis of pemphigus vulgaris does not involve apoptosis[14].Our results which included measurements of apoptosis markers and tissue staining exclude the role apoptosis in the pathogenesis of HHD. Staining of specimens of HHD with TUNEL staining in the involved areas of the tissues and the perilesional areas were devoid of apoptotic cells. Therefore, we hypothesize that reports suggesting a role for apoptosis may have shown apoptotic cells that are the result of the end process of acantholysis and not part of the initial phases related to the pathogenesis of HHD.

We next evaluated whether ATP2C1 plays any role in rat cardiomyocyte functioning or maintenance. Our assumption is based on two main reasons. The first is that the ATP2C1 protein is a part of Ca^2+^ signaling pathways that are indispensible for the heart contraction/relaxation processes. Second, it is well known that defects in ATP2C1 cause abnormal assembly of the desmosomes at level of the skin, though the exact mechanism is not yet clearly understood. The desmosomes also perform vital functions in the heart and several mutations in desmosomal proteins could cause either isolated cardiomyopathies[15,16], or cardiomyopathies in the setting of other skin manifestations[17]. One would thus hypothesize that a cardiac phenotype similar to that of the skin, would not be observed except under a stressful event in the setting of *ATP2C1* mutation. Such a phenotype could be a long recovery period or a worsening of a predicted event after cardiac-injury when compared to a normal, i.e. *ATP21C* mutation free setting. Interestingly, we have observed changes in transcript levels and protein levels at different time points during the heat-shock stressful experiment performed on cultured cardiomyocytes. Whether this increase in ATP2C1 plays some role in sustaining cardiomyocytes during stressful events could not be determined. Future experiments comparing wild type rat cardiomyocytes with mutants for *ATP2C1* would provide more data. Additionally, other experiments inducing cardiac stress such as mechanical cardiomyocyte stretching could be helpful. All together these results go in parallel with the role recently described for ATP2A2, a related member of the same family of Calcium dependent ATPases. In fact, numerous studies have established a strong role for this protein in cardiac function: mainly the cardiac-specific knock-out *ATP2A2* mice display cardiac mechanical and energetic dysfunctions [18,19]. Such phenotypes were only visualized in adult mice and under mechanical stretch conditions but were not observed in patients with Darier’s syndrome because the latter were not properly followed up. This is reminiscent of the lack of cardiac history among HHD patients, and our results suggest that mutations in *ATP21C* could lead to a cardiac adverse effect only following injury such as an infarct or in patients with chronic hypertension or diabetes. At that point it would be hard for the clinician to evaluate the contribution of any *ATP2C1* mutation to the ischemic event for instance, especially that many times HHD is not taken into consideration while treating such adverse events in these individuals. One way to better understand the role of ATP2C1 in cardiac patients would be a retrospective and a prospective study looking at individuals with HHD who had ischemic cardiac events and assess their clinical outcomes in terms of recovery as compared to the normal population.

In conclusion, this is the first genetic report of HHD in the Lebanese population, where we identified four different mutations in *ATP2C1* in five Lebanese families. Additionally, we demonstrated through *in vitro* studies a role for ATP2C1 in different skin cell types and found that apoptosis does not contribute to the pathogenesis of HHD. Moreover, we suggest that mechanisms other than haploinsufficiency could underlie HHD. Finally, we there might be a possible and still hidden role for ATP2C1 in cardiac function, where further work need to confirm and build on our early and preliminary data.
